# Mobile Health App Attitudes and Adoption Among Oncology Providers: Cross-Sectional National Survey

**DOI:** 10.2196/85583

**Published:** 2026-03-23

**Authors:** Nancy Lau, Kavin Srinakarin, Shannon JH Hong, Homer Aalfs, Michael E Roth, Karly M Ingram, Amy Berkman, Sherilynn Chan, Joanna Patten, Wade Iwata, Mallory R Taylor, Jesse R Fann, Eric J Chow, Tonya M Palermo

**Affiliations:** 1 Center for Child Health, Behavior and Development Seattle Children’s Research Institute Seattle, WA United States; 2 Department of Psychiatry and Behavioral Sciences University of Washington School of Medicine Seattle, WA United States; 3 Department of Psychology University of California, Berkeley Berkeley, CA United States; 4 Department of Pediatrics MD Anderson Cancer Center Houston, TX United States; 5 Department of Psychology East Carolina University Greenville, NC United States; 6 Department of Epidemiology and Cancer Control St Jude Children’s Research Hospital Memphis, TN United States; 7 Division of Psychiatry and Behavioral Medicine Seattle Children’s Hospital Seattle, WA United States; 8 Cancer and Blood Disorders Center Seattle Children’s Hospital Seattle, WA United States; 9 Ben Towne Center for Childhood Cancer and Blood Disorders Research Seattle Children’s Research Institute Seattle, WA United States; 10 Department of Pediatrics University of Washington School of Medicine Seattle, WA United States; 11 Clinical Research and Public Health Sciences Divisions Fred Hutch Cancer Center Seattle, WA United States; 12 Department of Anesthesiology & Pain Medicine University of Washington School of Medicine Seattle, WA United States

**Keywords:** digital health technology, digital health technologies, mobile application, mobile app, mobile health, mHealth, health personnel, oncology, psycho-oncology, delivery of health care, health care delivery

## Abstract

**Background:**

Mobile health (mHealth) apps can address health inequities and enhance access to care for individuals with immunocompromising conditions. Although hundreds of oncology apps exist, research on provider perspectives regarding their use in clinical care remains limited.

**Objective:**

This study aimed to describe oncology providers’ recommended apps, mHealth attitudes and beliefs, and perceived barriers to and facilitators of mHealth adoption. Exploratory aims examined differences based on provider type (medical vs psychosocial), provider age (<45 vs ≥45 years old), and patient population (pediatric vs adult).

**Methods:**

We conducted a cross-sectional survey administered via REDCap (Research Electronic Data Capture) to oncology providers across the United States between June and November 2024. Data were summarized using descriptive statistics. Pearson’s chi-square analyses examined exploratory group differences based on provider type, provider age, and patient age.

**Results:**

Of 188 respondents, the majority self-identified as female (150/188, 79.8%), White (161/188, 85.6%), and non-Hispanic/Latino (174/188, 92.6%). Nearly all providers (178/188, 94.7%) reported either recommending or using mHealth apps with their patients, with primary use for patient-provider communication (139/188, 73.9%). Providers perceived potential benefit across a broad spectrum of holistic care functions. Providers, on average, reported a growth mindset and confidence in their ability to learn mHealth tools and in its potential to improve care access. Key facilitators included alignment with patient needs, increased accessibility, and cost-effectiveness, while barriers included disparities in technology access, digital health literacy, and data security and privacy. Exploratory analyses showed some significant group differences by provider role, provider age, and patient age. Psychosocial providers were significantly more likely to recommend or use apps for pain management (*χ*^2^_1_=14.34, *P*<.001, φ=0.28), mental health (*χ*^2^_1_=50.54, *P*<.001, φ=0.53), and sleep health (*χ*^2^_1_=25.47, *P*<.001, φ= 0.38). Psychosocial providers also perceived higher benefit for sleep health apps (*χ*^2^_1_=6.40, *P*=.01, φ=0.19). Medical providers were significantly more likely to perceive medication management apps as potentially beneficial (*χ*^2^_1_=10.93, *P*<.001, φ=0.25). Older providers (16/88, 18.2%) and adult care providers (8/32, 25%) were significantly more likely to recommend or use disease management apps compared to younger providers (5/100, 5%; *χ*^2^_1_=8.20, *P*=.004, φ=0.21) and pediatric care providers (6/101, 5.9%; *χ*^2^_2_=9.22, *P*=.01, Cramer V=0.22), respectively. Pediatric care providers (83/101, 82.2%) were more likely to recommend or use medical team communication apps compared to adult care providers (15/32, 46.9%; *χ*^2^_2_=15.66, *P*<.001, Cramer V=0.29).

**Conclusions:**

Our study underscores the opportunity to develop inclusive mHealth solutions tailored to the diverse needs of individuals across the cancer care continuum, including those in active treatment and survivorship care. Engaging diverse medical and psychosocial providers is essential to inform clinical integration of mHealth technologies in oncology care.

## Introduction

Over the past decade, the worldwide adoption and use of smartphone technologies has grown exponentially. The total number of smartphone users exceeds 6.7 billion worldwide [1]. Smartphones have become ubiquitous, regardless of gender, race, ethnicity, level of education, and socioeconomic status [2-5]. Over 350,000 mobile health (mHealth) apps (software apps downloadable on mobile devices) are available for public download on Google Play and Apple App Store [6]. mHealth apps have the potential to reduce health disparities for underresourced populations and to improve accessibility for those with immunocompromised or debilitating health conditions [7-15] .mHealth apps may perform a wide range of health care delivery and information dissemination functions.

A 2016 systematic review identified 539 mHealth oncology apps available for public download [16]. Content categories across the oncology apps included teaching and information, medication help, monitoring, prevention, screening, smoking cessation, and patient support. A more recent 2021 systematic review identified 794 mHealth oncology apps available for public download [17], a 47% increase in new apps created over a 5-year time frame. Content categories across oncology apps included education, clinical decision support, and patient support. However, most available oncology apps have not been developed or tested in research settings, and few have Food and Drug Administration (FDA) approval as “digital therapeutics” [16-23]. A 2020 systematic review identified only nine publicly available oncology apps with scientific publications supporting their feasibility or efficacy, suggesting an industry-research gap [18].

Recent research suggests that patients with cancer are interested in using mHealth apps. Studies have found positive patient attitudes toward mHealth apps for a range of functions over the cancer care continuum [24-27], such as medication adherence, early detection and diagnostics, patient-provider communication, lifestyle interventions, weight and diet management, exercise facilitation, symptom monitoring, medical health literacy, disease self-management, and medical decision-making [28-38]. Results of a 2018 oncology patient survey showed that over 60% of survey respondents regularly used the internet to research information about disease prevention, healthy lifestyles, health care, and their cancer treatment [39]. Over 80% of respondents stated that they would download and use an app if their health care provider recommended it to them, highlighting the importance of health care provider involvement in patient adoption of mHealth tools [39].

To date, relatively few studies have examined oncology health care provider attitudes toward mHealth apps [21,40,41]. An international survey of oncologists showed that 80% of respondents used mHealth, primarily for the following functions: to conduct literature reviews, to interact with colleagues, for patient communication, to access patient electronic health records, to collect test results, and for follow-up care [40]. A single-site provider survey of oncologists and nurses in Germany found that the mHealth functions rated as most useful were appointment and medication reminders, assessment of side effects, and collecting patient-reported outcomes [41]. Most survey respondents considered mHealth apps to be a welcome complement to traditional health care and perceived benefits to implementing mobile apps as part of the standard of care. Provider concerns endorsed by a minority of respondents included legal/medical responsibility, data privacy, and data security. A qualitative interview study [21] found that oncology providers expressed openness to mHealth apps and acknowledged their potential to fill care gaps. Perceived implementation barriers included limited access to technologies, time burden, and concerns about the trustworthiness and credibility of app developers [21]. Providers prioritized app usability and patient preferences over app efficacy or effectiveness [21].

Early research on provider attitudes toward mHealth apps focused primarily on oncologist perspectives and disease management functions. Here, we filled a research gap by incorporating perspectives from providers across diverse health care roles, including medical and psychosocial providers. We included providers who work with patients across the age continuum who may have different attitudes toward and familiarity with mHealth. We explored feedback on mHealth content that extends beyond disease management to include behavioral health and holistic patient care. Holistic patient care is a “whole person” approach to medicine that encompasses physical, mental, emotional, lifestyle, and social needs—not just treating symptoms of disease—in order to promote overall well-being and improve long-term health outcomes [42-44]. This broader focus is critical for understanding how technology can support a more comprehensive approach to patient outcomes. Consequently, this study sought to report an update and extension of prior research by answering the following research questions (RQs): (1) What types of apps have oncology providers used with their patients or would consider potentially beneficial? (2) What are the attitudes, beliefs, facilitators, and barriers related to the adoption of mHealth apps in oncology settings? (3) (Exploratory) How do provider preferences differ based on provider role (medical vs psychosocial), provider age (<45 vs ≥45 years), and patient populations served (pediatric vs adult)? The selection of exploratory variables was informed by existing mHealth and technology acceptance literature [45-50]. Findings will inform recommendations for apps that providers perceive to have clinical utility, future directions for app development, and methods to promote implementation and uptake of mHealth apps.

## Methods

### Ethical Considerations

All study procedures were approved by the Seattle Children’s Hospital Institutional Review Board (IRB approval number STUDY00004962; approval date June 11, 2024). The study was conducted under IRB-exempt status in which survey information obtained is recorded in a manner that the identity of the survey respondents cannot be ascertained. Survey participation was voluntary and anonymous, and no compensation was offered for completing the survey. A one-page REDCap (Research Electronic Data Capture) Study Information Sheet containing purpose of the study, investigator information, study description, confidentiality statement, data storage details, and contact information was provided on the main screen of the online questionnaire. Survey respondents were informed via the Study Information Sheet that their responses would remain anonymous and research records would be stored exclusively within secure institutional intranet databases. The survey was administered through Seattle Children’s Hospital REDCap, a secure web-based survey platform that uses password-protected access and role-based permissions to prevent unauthorized data access.

### Study Design

We disseminated an online survey called the “Oncology Provider Experiences National Survey (OPENS): Mobile Health” (hereafter referred to as “OPENS”) requesting responses on provider attitudes and experiences toward the use of mHealth apps in oncology. The target population for this study consisted of health care professionals currently practicing in oncology settings within the United States. Participation was open to all who self-identified as an oncology provider. Although we did not include an eligibility screener, eligibility was controlled with targeted recruitment through oncology provider list servers (listservs). Our survey was distributed nationally across the United States to members of the American Psychosocial Oncology Society (APOS) and provider listservs at the home institutions of several of the coauthors (eg, Seattle Children’s Hospital Cancer and Blood Disorders Center, Fred Hutchinson Cancer Center, MD Anderson Cancer Center, St Jude Children’s Research Hospital). We distributed the survey to several organizations that are pediatric-focused: Children’s Oncology Group (COG) Behavioral Science Committee, Society of Pediatric Psychology Hematology/Oncology/Bone Marrow Transplant Special Interest Group, and Association of Pediatric Oncology Social Workers (APOSW). Permission to distribute our online survey was obtained by research committees or administrators, as appropriate for each respective listserv. Each listserv was sent a single request with a designated survey end date and with no follow-up reminders. We staggered survey distribution pending submission and review of materials by each listserv’s respective research committee and administrators. Prospective participants were informed via the survey dissemination email and the REDCap Study Information Sheet that the survey was intended for oncology providers only. The survey took approximately 10 minutes to complete. The survey remained open from June 2024 to December 2024. This study was reported in accordance with CHERRIES (Checklist for Reporting Results of Internet E-Surveys). See Multimedia Appendix 1 for the complete checklist.

### Oncology Provider Experiences National Survey: Mobile Health

To the best of our knowledge, no validated measure currently exists to assess oncology provider attitudes toward mHealth apps. We developed the 30-item OPENS to describe mHealth-related usage, attitudes, perceived benefit, facilitators, and barriers. OPENS was designed as a descriptive survey to assess provider perspectives and usage patterns rather than as a psychometric scale intended to measure a single latent construct. These survey items directly address our study aims to inform the clinical integration of mHealth technologies in oncology care. Internally developed surveys are a standard approach in digital health research when established measures are unavailable [51-54]. Survey items were collaboratively developed by a multidisciplinary research team (including experts in psychology, psychiatry, social work, oncology, and digital health) following a targeted literature review. We established content validity using a modified Delphi approach [55-58]. This process involved asynchronous feedback in which collaborators independently reviewed survey items for clarity and relevance to both the study aims and the oncology clinical context. Facilitated group consensus meetings were held with external oncology experts to refine survey items. Eight items assessing mHealth attitudes and beliefs were adapted from the “Questionnaire to Assess Public Health Staff Viewpoints about Mobile Phone Use in the Delivery of Their Services” [59]. These items were updated for oncology relevance. Survey items were finalized asynchronously among the research team members.

Before national dissemination, the survey underwent pilot-testing with a small group of oncology providers to assess technical functionality within REDCap, item comprehension, and user burden. All 33 items were distributed across 4 pages and administered in a forced-response format to ensure complete data capture. Questions included single-choice items, multiple-choice items, “5-point Likert scale” items (eg, strongly disagree to strongly agree), and “select all that apply” items (including “none of the above” and “other” options). Respondents were able to review and modify their responses prior to survey submission, with no time limits or automatic expiration imposed for completion. Consistent with the scoring method of the validated instrument from which eight survey items were adapted [48], Likert-scale responses were summarized using means (SDs) to characterize the strength and variability of providers’ beliefs and attitudes on mHealth apps. The brief close-ended survey was designed to reduce participant burden and maximize response rates for uncompensated national listserv recruitment methods, targeting a completion time of approximately 10 minutes. To ensure that our study met IRB-exempt criteria, we did not include open-ended survey questions in which human subjects’ identifying information may have inadvertently been obtained. See Multimedia Appendix 2 for all survey items.

### Statistical Analysis

We summarized provider demographics and other survey items using descriptive statistics and data visualizations created in Canva. Exploratory statistical analyses were performed using IBM SPSS Statistics (version 29). Study results are presented as frequency counts, percentages, and means (SDs). We also conducted exploratory analyses on survey responses broadly categorized by provider type (medical providers vs psychosocial providers) who may differ in their attitudes toward and the use of mHealth tools for disease management and behavioral/emotional support based on their training backgrounds and areas of expertise. For the purposes of this study, medical providers were defined as those whose primary training and clinical focus address disease treatment and the management of physiological symptoms. This group included those who identified their role as oncologist, nurse or advanced practice provider, physician, or health care administrator. Psychosocial providers were defined as clinicians whose primary training and clinical focus address the psychological, social, and emotional aspects of the cancer experience. This group included those who identified their role as psychologist, social worker, mental health therapist, or occupational therapist. This categorization also allowed for a balanced distribution of providers for comparison (90/188, 47.9%, medical vs 89/188, 47.3%, psychosocial). We performed chi-square analyses to examine differences between medical providers and psychosocial providers on the use of mHealth apps in clinical practice, mHealth apps endorsed as potentially helpful or beneficial, and the perceived facilitators of and barriers to mHealth app adoption. We also performed chi-square analyses to examine differences between younger providers (age≤45 years) vs older providers (age≥46 years) and pediatric care providers (children, teens, or young adults) vs adult care providers (adults, older adults) on the use of mHealth apps in clinical practice. Given that the study used categorical data, traditional assumptions of normality were not applicable. As all chi-square analyses were exploratory and intended for hypothesis generation rather than formal hypothesis testing, we did not control for multiple comparisons.

## Results

### Demographic and Provider Role Characteristics

A total of 188 providers responded to the survey (Table 1). Participants ranged in age from under 24 to over 65 years. Over one-third (70/188, 37.2%) were between the ages of 36 and 45 years. Most providers identified as female (150/188, 79.8%), White (161/188, 85.6%), and not Hispanic or Latino (174/188, 92.6%).

**Table 1:** Demographic characteristics of oncology providers (N=188).

Characteristics		Respondents, n (%)
**Age group (years)**		
	<24	2 (1.1)
	24-35	28 (14.9)
	36-45	70 (37.2)
	46-55	42 (22.3)
	56-65	29 (15.4)
	>65	17 (9.0)
**Gender identity**		
	Female	150 (79.8)
	Male	37 (19.7)
	Prefer not to respond	1 (0.5)
**Race**		
	American Indian or Alaska Native	2 (1.1)
	Asian	13 (6.9)
	Black or African American	0 (0.0)
	Hispanic or Latino	7 (3.7)
	Middle Eastern or North African	1 (0.5)
	Native Hawaiian or Pacific Islander	0 (0.0)
	White	161 (85.6)
	Not listed	2 (1.1)
	Prefer not to respond	4 (2.1)
**Ethnicity**		
	Hispanic or Latino	12 (6.4)
	Not Hispanic or Latino	174 (92.6
	Prefer not to respond	2 (1.1)

Of the 188 respondents, 47.9% (90/188) were medical providers, 47.3% (89/188) were psychosocial providers, and 4.8% (9/188) had administrative staff or research roles. Psychologists represented the largest group of survey respondents (66/188, 35.1%), followed by oncologists (54/188, 28.7%) and nurses or advanced practice providers (32/188, 17%), as shown in Table 2. Most providers practiced in academic or teaching hospitals (155/188, 82.4%) and urban settings (130/188, 69.1%). Over half of the sample provided care for children (120/188, 63.8%), teens (124/188, 66%), and young adults (143/188, 76.1%).

**Table 2:** Role characteristics of oncology providers (N=188).

Characteristics		Respondents, n (%)
**Current role^a^**		
	Oncologist	54 (28.7)
	Nurse or advanced practice provider	32 (17.0)
	Psychologist	66 (35.1)
	Social worker	13 (6.9)
	Health care administrator	2 (1.1)
	Researcher	33 (17.6)
	Physician (other specialty)	4 (2.1)
	Other	14 (7.4)
**Practice setting^a^**		
	Private practice	11 (5.9)
	Academic or teaching hospital	155 (82.4)
	Community hospital	16 (8.5)
	Hospice care	0 (0.0)
	Other	15 (8.0)
**Practice location**		
	Rural	15 (8.0)
	Suburban	35 (18.6)
	Urban	130 (69.1)
	Other	8 (4.3)
**Duration of experience providing care (years)**		
	<1	1 (0.5)
	1-5	39 (20.7)
	6-10	41 (21.8)
	11-15	38 (20.2)
	>16	68 (36.2)
	Prefer not to respond	1 (0.5)
**Patient age demographic^a^**		
	Children	120 (63.8)
	Teens	124 (66.0)
	Young adults	143 (76.1)
	Adults	82 (43.6)
	Older adults	50 (26.6)

^a^Questions on the respondents’ current role, practice setting, and patient age demographic were multiple-response items; providers could select all applicable options.

### mHealth Apps Used in Clinical Practice

**Figure 1:**
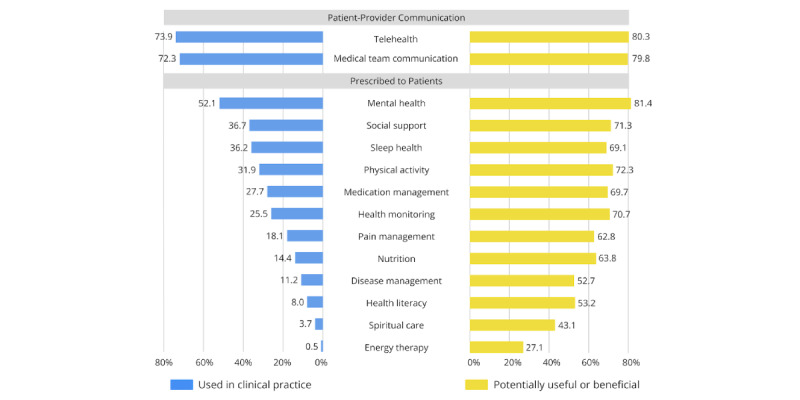
Comparison of mHealth apps used in clinical practice vs potentially useful or beneficial mHealth apps for patients. mHealth: mobile health.

**Figure 2:**
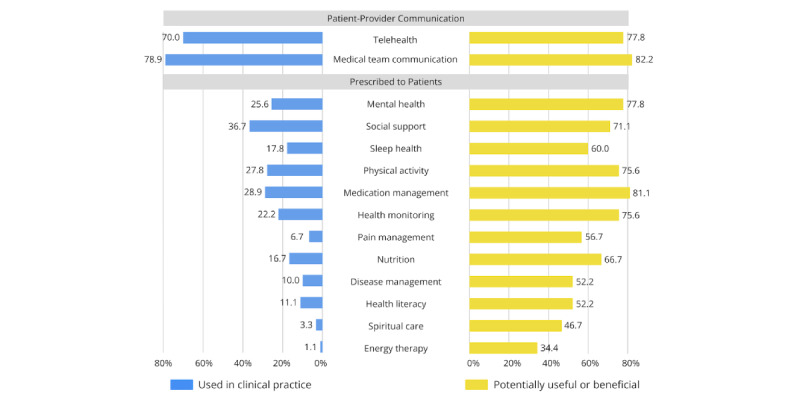
Comparison of mHealth apps used in clinical practice vs potentially useful or beneficial mHealth apps for medical providers. mHealth: mobile health.

**Figure 3:**
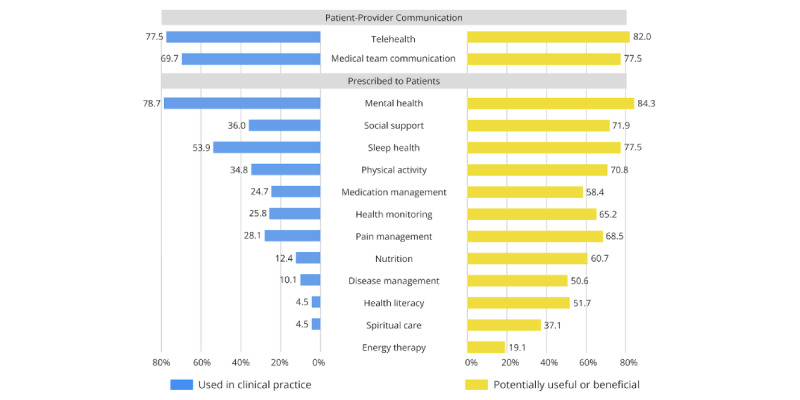
Comparison of mHealth apps used in clinical practice vs potentially useful or beneficial mHealth apps for psychosocial providers. mHealth: mobile health.

Nearly all providers (178/188, 94.7%) reported recommending or using at least one mHealth app with their patients (Figure 1). Most providers recommended or used telehealth and virtual consultation apps (139/188, 73.9%). Other types of mHealth apps that were commonly recommended to or used with patients included communication with the medical team (136/188, 72.3%) and mental health and wellness (98/188, 52.1%) apps. Only a small proportion of providers reported recommending or using cancer and disease management (21/188, 11.2%) or health literacy (15/188, 8%) mHealth apps with their patients. Both medical and psychosocial providers predominantly used mHealth apps for medical team communication or telehealth (Figure 2 and Figure 3, respectively). Psychosocial providers (25/89, 28.1%) were significantly more likely than medical providers (6/90, 6.7%) to recommend or use pain management apps (*χ*^2^_1_=14.34, *P*<.001, φ=0.28). Psychosocial providers (70/89, 78.7%) were significantly more likely than medical providers (23/90, 25.6%) to recommend or use mental health apps (*χ*^2^_1_=50.54, *P*<.001, φ=0.53). Psychosocial providers (48/89, 53.9%) were significantly more likely than medical providers (16/90, 17.8%) to recommend or use sleep health apps with their patients (*χ*^2^_1_=25.47, *P*<.001, φ=0.38). All other chi-square analyses comparing medical and psychosocial provider groups were nonsignificant.

Providers stratified by age (≤45 and ≥46 years) recommended or used similar types of mHealth apps with patients (Multimedia Appendix 3). Older providers (16/88, 18.2%) were significantly more likely than younger providers (5/100, 5%) to recommend or use disease management apps with their patients (*χ*^2^_1_=8.20, *P*=.005, φ=0.21). All other chi-square analyses comparing older and younger providers were nonsignificant. Providers working with pediatric and adult patients also recommended or used similar types of mHealth apps (Multimedia Appendix 4). Adult care providers (8/32, 25%) were significantly more likely than pediatric care providers (6/101, 5.9%) to recommend or use disease management apps (*χ*^2^_2_=9.22, *P*=.01, Cramer V=0.22). Pediatric care providers (83/101, 82.2%) were more likely than adult care providers (15/32, 46.9%) to recommend or use medical team communication apps (*χ*^2^_2_=15.66, *P*<.001, Cramer V=0.29). All other chi-square analyses comparing adult and pediatric care providers were nonsignificant.

### Potentially Useful or Beneficial mHealth Apps

All providers endorsed mHealth apps to be potentially useful or beneficial for patients (Figure 1). Most providers perceived mental health and wellness apps to be potentially useful (153/188, 81.4%). Other types of mHealth apps that were perceived as potentially useful were telehealth and virtual consultation (151/188, 80.3%), communication with the medical team (150/188, 79.8%), exercise and physical activity (136/188, 72.3%), social support (134/188, 71.3%), health monitoring and medical symptom tracking (133/188, 70.7%), medication management (131/188, 69.7%), and sleep health (130/188, 69.1%). Although providers predominantly used mHealth apps for patient communication (eg, medical team communication, telehealth), they endorsed interest in prescribing mHealth apps for a broad range of holistic care functions.

Interest in a wide array of psychosocial support functions was fairly consistent across medical and psychosocial providers (Figure 2 and Figure 3, respectively). Medical providers (73/90, 81.1%) were significantly more likely than psychosocial providers (52/89, 58.4%) to perceive medication management apps as potentially beneficial for their patients (*χ*^2^_1_=10.93, *P*<.001, φ=0.25). Medical providers (31/90, 34.4%) were significantly more likely than psychosocial providers (17/89, 19.1%) to perceive energy therapy apps as potentially beneficial (*χ*^2^_1_=5.37, *P*=.02, φ=0.17). In contrast, psychosocial providers (69/89, 77.5%) were significantly more likely than medical providers (54/90, 60%) to endorse sleep health apps as potentially helpful for their patients (*χ*^2^_1_=6.40, *P*=.01, φ=0.19). All other chi-square analyses comparing medical and psychosocial provider groups were nonsignificant.

### mHealth Attitudes and Beliefs

Providers had positive attitudes and beliefs toward specific facets of mHealth apps (Table 3). Providers had the highest mean scores for specific items pertaining to health equity, indicating that they “agree” or “strongly agree” that mHealth apps reduce travel costs for services (mean 4.18/5, SD 0.68) and expands the range of health services (mean 4.18/5, SD 0.69). Providers had the highest mean scores for specific items pertaining to confidence and a growth mindset, indicating that they “agree” or “strongly agree” that they are confident in their ability to navigate mobile apps (mean 4.14/5, SD 0.83) and can adapt to health technology changes over time (mean 4.18/5, SD 0.70). Providers had the lowest mean scores for specific items pertaining to the patient-provider relationship and training, indicating that they “neither agree nor disagree” that mHealth improves provider-patient interactions (mean 3.21/5, SD 0.95) or are trained and informed about mHealth regulatory and ethical issues in health care (mean 3.24/5, SD 1.07).

**Table 3:** Attitudes and beliefs toward mHealth^a^ apps among oncology providers.

Item		Mean (SD)
**Training**		
	I feel that I am trained and informed about regulatory and ethical issues related to technology use in health care.	3.24 (1.07)
	I can effectively teach patients how to use mHealth technologies for their care.	3.44 (1.03)
	I am interested in receiving more training or specialized training in the use of digital tools in my health care practice.	3.88 (0.96)
**Confidence and growth mindset**		
	I can adapt to health technology changes over time.	4.18 (0.70)
	I feel confident in my ability to navigate mobile apps.	4.14 (0.83)
	I am comfortable incorporating technology into patient care practices.	3.74 (0.90)
**Quality of care**		
	The use of mobile technology improves the quality of public health services.	3.92 (0.68)
	Mobile technology can help improve patients’ quality of life.	3.94 (0.66)
	Mobile technology improves self-management in public health care professionals.	3.65 (0.76)
	Mobile technology can improve patients’ health literacy and encourage them to actively engage in their care.	3.86 (0.73)
**Health equity**		
	Using mobile technology reduces the cost of providing public health services.	3.64 (0.83)
	The use of mobile technology expands the range of health services.	4.18 (0.69)
	The use of mobile technology creates equal access to facilities and services for the general public.	3.31 (0.98)
	Using mobile technology reduces the number of visits to health centers.	3.77 (0.83)
	The use of mobile technology reduces travel costs for services.	4.18 (0.68)
**Patient-provider relationship**		
	Mobile technology improves interpersonal interactions between the provider and the recipient.	3.21 (0.95)
	Integrating mobile technology can improve patient-provider interactions.	3.81 (0.70)

^a^mHealth: mobile health.

### Facilitators of mHealth Adoption

**Figure 4:**
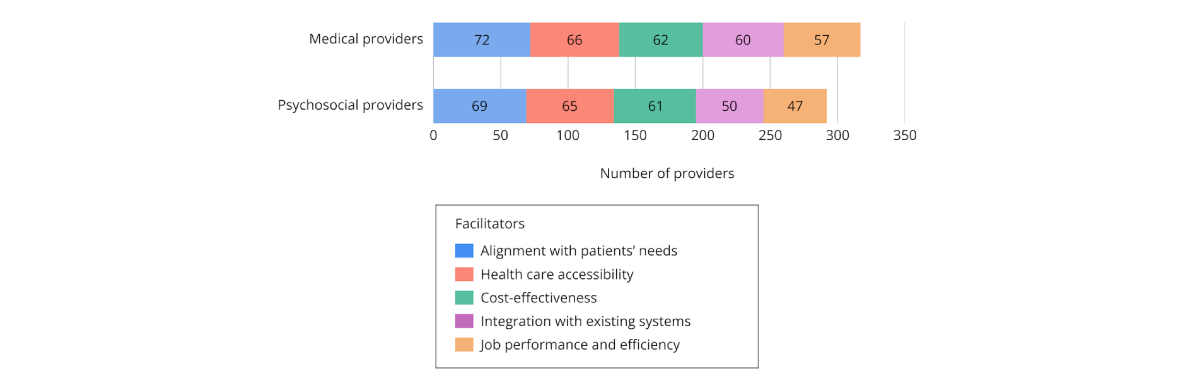
Common facilitators of adopting mHealth apps in health care settings among medical and psychosocial providers. mHealth: mobile health.

The top five perceived facilitators of mHealth adoption in clinical settings were “alignment with patients’ expressed interest and needs” (147/188, 78.2%), “improved health care accessibility” (135/188, 71.8%), “cost-effectiveness for patients and health care systems” (127/188, 67.6%), “seamless integration with existing health care systems and/or electronic medical records” (113/188, 60.1%), and “improved job performance and efficiency” (109/188, 58%); see Multimedia Appendix 5. Medical and psychosocial providers reported similar top facilitators overall (Figure 4). However, medical providers (47/90, 52.2%) were significantly more likely than psychosocial providers (23/89, 25.8%) to endorse patient health literacy as a perceived facilitator (*χ*^2^_1_=13.08, *P*<.001, φ=0.27).

### Barriers to mHealth Adoption

**Figure 5:**
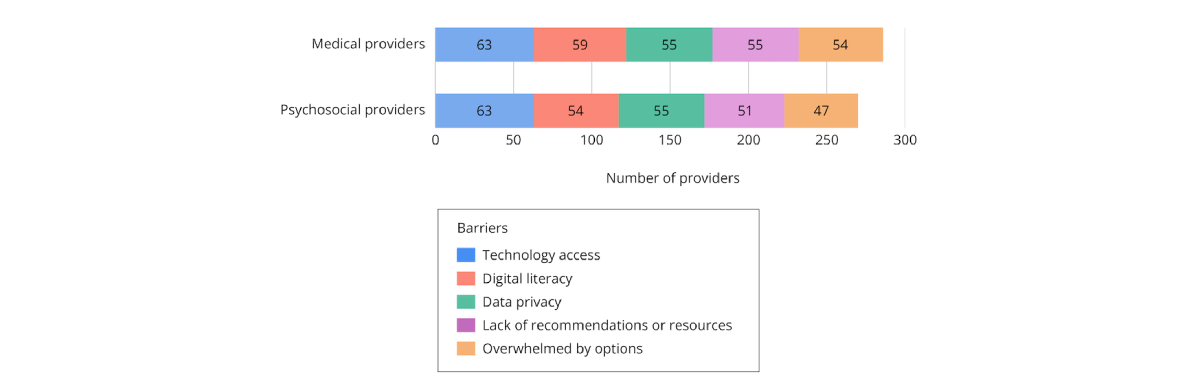
Common barriers to adopting mHealth apps in health care settings among medical and psychosocial providers. mHealth: mobile health.

The top five perceived barriers to mHealth adoption in clinical settings were “disparities in access to technology” (132/188, 70.2%), “digital literacy” (ie, technical knowledge to access, understand, and use digital resources and tools; 117/188, 62.2%), “data privacy and safety concerns” (114/188, 60.6%), “lack of knowledge regarding which digital tools to recommend or where to find relevant information” (110/188, 58.5%), and “being overwhelmed by too many digital health technology options” (105/188, 55.9%); see Multimedia Appendix 6. Medical and psychosocial providers reported similar top barriers overall (Figure 5). However, medical providers (52/90, 57.8%) were more likely than psychosocial providers (38/89, 42.7%) to endorse cost as a perceived barrier (*χ*^2^_1_=4.07, *P*=.04, φ=0.15).

## Discussion

### Principal Findings

This study provided an update to and extension of prior research regarding the integration of mHealth apps in oncology care. Regarding mHealth app usage and its perceived benefit (RQ1), oncology providers commonly use mHealth apps, with an emphasis on patient-provider communication tools. Although current usage is primarily logistical, there is clear interest in whole person health–supporting apps that providers consider potentially beneficial but that currently see lower rates of adoption. With regard to provider attitudes, facilitators, and barriers related to mHealth adoption (RQ2), providers consistently endorsed a growth mindset, reporting high confidence in their ability to learn and adopt digital tools. Providers recognized the potential for mHealth to improve health equity by expanding access to care. The top perceived facilitators of mHealth adoption were alignment with patient needs, improved health care accessibility, and cost-effectiveness. The top perceived barriers to mHealth adoption were disparities in access to technology, digital health literacy, and data privacy.

With regard to exploratory differences by clinical role, provider age, and patient age (RQ3), we found several group differences. Reflecting their respective training backgrounds, medical providers were more likely to endorse the potential benefit of medication management apps, whereas psychosocial providers were more likely to use and/or perceive potential benefit in pain management, mental health, and sleep health apps. The use of mHealth apps was generally consistent across oncology provider age groups (≤45 and ≥46 years) and patient population (pediatric care providers and adult care providers). However, older providers (age≥46 years) and those providing care to adult patients used more disease management tools, whereas pediatric providers used more medical team communication apps.

### Comparison With Prior Work

Our study expands on the background literature on oncology providers’ attitudes toward mHealth apps by soliciting perspectives of those in various clinical roles, not exclusively oncologists; expanding mHealth app functions beyond disease management; and identifying perceived barriers to and facilitators of implementation in oncology care settings. Our findings are consistent with previous studies that have reported that oncologists primarily use mHealth tools for telehealth and medical team communication and rarely use mHealth tools for disease management and follow-up care [40]. Of note, we also found that medical and psychosocial providers alike are interested in using mHealth apps focusing on a wide range of psychosocial care functions to their patients and perceived them to have clinical utility. This was consistent with the literature on patients with cancer and those who have survived cancer, showing high rates of acceptance of mHealth apps but low rates of adoption [60-64]. Specific patient facilitators and barriers beyond demographic and disease characteristics have not yet been examined. Studies examining patient preferences have predominantly focused on disease management apps [60,62-66]. Consistent with the background literature, we found that a primary barrier identified by providers is disparities in access to technology [21,40,67]. However, research has shown that mHealth technologies are universally accessible to individuals regardless of income level, rurality, or marginalized status [3,4,68]. In the United States, 79%-94% of individuals from low-income households (<US $30,000) have access to smartphones, computers, and tablets [3,4,69]. Digitally deployed health interventions have also been proposed for populations/persons experiencing homelessness as viable alternatives to in-person services to overcome traditional access care barriers [68,70,71]. In the United States, 91% of individuals own a smartphone, 95% use the internet, and 90% have a high-speed internet subscription at home [1,3,4,69]. Universal use of smartphones holds across the age continuum, with 95% of teens/young adults, 95%-97% of adults aged>45 years [2-4,72], and 76%-79% of older adults aged>65 years owning a smartphone [4,69,72]. Digital technologies (smartphones, computers, tablets) have become significantly more affordable in recent years, and their rapid adoption has been further accelerated by the COVID-19 pandemic.

Consistent with the background literature, a primary barrier to mHealth adoption, as identified by providers, was limited digital health literacy [61]. Previous studies have shown that health literacy and digital health literacy are significant mediators of the efficacy of digital health interventions [73] and are not fixed but malleable patient characteristics [74]. Thus, providers may be more likely to recommend and use mHealth apps with intuitive user interfaces, accessibility for those with impairments, provision of health information at lower reading levels, and multimedia options (eg, text-to-speech and animations) [61,75,76]. Recommendations for mHealth may also be paired with apps that identify a user’s health literacy level and provide tailored definitions of medical terminologies to their reading level (eg, MediReader [77]).

Previous research has found that younger oncologists (<45 years old) and younger cancer patients and survivors (<45 years old) are more likely to use internet-enabled mHealth tools for disease management and cancer care [40]. Our results showed consistent use of mHealth apps in younger as well as in older providers (>45 years old) and among providers of patients across the age continuum. For younger pediatric patients, caregivers may primarily interact with mHealth apps, while teens and young adults may be more independent app users [78]. Digital inclusion for older adults is recognized as an important social determinant of health and a crucial component of a healthy aging society [79-88]. These findings highlight the importance of developing, testing, and implementing mHealth apps that are tailored to the diverse needs of patients with cancer and those who have survived cancer, of all ages.

The Technology Acceptance Model (TAM) proposes that perceived usefulness (ie, the belief that using a particular technology enhances job performance) and perceived ease of use (ie, the belief that using a particular technology is effort free) are the primary drivers of user adoption of new technologies [89,90]. Interpreted through the TAM, our findings suggest high readiness for mHealth adoption across the oncology workforce. Specifically, providers’ endorsement of mHealth apps for reducing costs and expanding care access aligns with high perceived usefulness. Providers’ reported growth mindset and confidence in their ability to learn and adapt to mHealth technologies reflect high self-efficacy, which aligns with high perceived ease of use. Although TAM pathways were not formally tested in our study, the alignment between our findings and these TAM constructs suggest that oncology providers have the positive attitudes and behavioral intentions necessary to facilitate mHealth adoption.

### Future Directions

mHealth apps that address holistic patient care have the potential to improve patient outcomes across the cancer care continuum and patient age continuum. Despite the narrow use of mHealth apps in current practice, the perceived utility of mHealth across medical and psychosocial domains suggests important opportunities for broader integration. Guided by the RE-AIM (Reach, Effectiveness, Adoption, Implementation, and Maintenance) framework [91-93], future research should focus on expanding *reach* by partnering with medical and psychosocial providers on codesign strategies of mHealth tools in the innovation design-to-implementation pipeline. Future research should also enhance *effectiveness* via rigorous clinical testing and addressing the complex ethical and regulatory landscape of mHealth apps. Although oncology providers are interested in using mHealth tools, the lack of standardized clinical guidelines for recommending non-FDA-approved apps remains an important barrier. Endorsing or recommending mHealth clinical tools that lack rigorous scientific testing poses risks to patient safety and data confidentiality. Future research and policy work should focus on developing clinical guidelines to identify evidence-based mHealth apps and pathways for determining their clinical efficacy/effectiveness. In addition, future research should support *adoption* and *implementation* by examining providers’ nuanced perspectives on the perceived barrier of technology access and investigate the specific sources of these concerns (eg, financial burden of cancer, lack of reliable cellular or internet service in rural areas). Efforts to mitigate barriers, such as concerns about data privacy and security, will also be critical to ensuring adoption of mHealth solutions. Successful integration of mHealth apps into oncology care will require accessible, user-centered design (eg, intuitive interfaces, appropriate reading levels, multimedia supports) and shared decision-making informed by ongoing examination of concordant and discordant beliefs and attitudes toward mHealth apps among providers and patients. Long-term *maintenance* will depend on sustained institutional and policy support (reimbursement pathways, standardized evaluation criteria, regulatory guidance on data privacy and security), engagement of local providers or stakeholder champions to promote uptake through education and shared learning in navigating digital health systems, and preservation of alternative care options (eg, in-person services) as technologies continues to evolve. Future research using a mixed methods approach is also warranted to provide richer context to these exploratory findings.

### Limitations

This study has several limitations. First, this is a convenience sample of oncology providers, which introduces the potential for selection and response bias due to voluntary participation. It is possible that providers with a pre-existing interest in mHealth were more motivated to participate in the study, potentially leading to nonresponse bias where favorable attitudes are overrepresented. In addition, social desirability bias may have influenced participants to overreport favorable attitudes to align with current digital health trends. To mitigate potential bias, we distributed the survey through multiple national professional listservs representing diverse professional roles. In addition, survey questions assessed diverse app functions, barriers, and facilitators, which resulted in variability in study findings. This suggests that the survey captured an array of provider perspectives. Second, most respondents identified as White and female, practiced in urban settings, and did not see older adults in their clinical practice. This limits the generalizability and transferability of our findings to underrepresented providers because of a lack of diversity in our sample. Future studies should implement recruitment strategies to target provider demographic diversity, rural health settings, and those in older adult and geriatric care to ensure findings are representative of all clinical contexts. Third, this is a small sample relative to the number of oncology providers who practice nationally, and we relied on our institutional and national professional organization listservs to distribute the survey. Sample size was determined by convenience sampling within the study recruitment time frame. As the primary aim of this study was descriptive, we did not establish a predetermined target sample size. Fourth, as there are no well-validated measures of provider attitudes toward mHealth interventions with established cutoff scores, we used an internally developed survey. This influenced the study by limiting our ability to categorize and compare scores with existing studies. To mitigate the lack of a pre-existing validated scale, we used a modified Delphi process involving a multidisciplinary team and external oncology experts to ensure content validity. Fifth, to comply with IRB-approved protocols and IRB-exempt status, we did not collect identifiers from survey respondents, so it is not possible to determine response rates from specific organizations or listservs or analyze group differences based on affiliations. Future research could include such identifiers in order to assess representativeness and potential cohort effects. Sixth, due to the exploratory nature of our analyses, we did not control for multiple comparisons or include demographic covariates. Although this approach increases the risk of type 1 errors, we mitigated the risk of overinterpretation by explicitly framing these analyses as hypothesis generating, and our findings should be viewed as preliminary. Finally, this was a cross-sectional study who findings provide a snapshot in time of provider attitudes in a post-COVID-19 pandemic landscape that has normalized digital health care. Because attitudes and perspectives may continue to shift over time and technology evolves rapidly, future longitudinal research is warranted.

### Conclusion

The findings of this study reveal a significant “readiness gap” in oncology care. Although current use remains largely focused on telehealth services and patient-provider communication, providers expressed interest in integrating a wider array of mHealth apps encompassing health information, disease and symptom monitoring, mental health resources, and psychosocial support. Our findings suggest that the oncology workforce is prepared to move beyond logistical communication tools toward an integrated holistic model of oncology care that encompasses broad disease management and psychosocial support functions. Because these positive attitudes are consistent across clinical roles and patient populations, mHealth interventions have broad appeal and should be implemented as interdisciplinary standards of care. Health care systems can leverage existing provider buy-in to create a more equitable and accessible oncology care model.

## Data Availability

The study was conducted under Institutional Review Board (IRB)-exempt status, and data generated are not publicly available. Data may be made available from the corresponding author (NL) upon reasonable request and subject to a formal data use agreement.

## Appendix

Multimedia Appendix 1 Checklists for Reporting Results of Internet E-Surveys (CHERRIES).

Multimedia Appendix 2 Oncology Provider Experiences National Survey (OPENS): Mobile Health.

Multimedia Appendix 3 Types of mobile health (mHealth) apps providers prescribed to or used with patients stratified by provider age (≤45 vs ≥46 years).

Multimedia Appendix 4 Types of mobile health (mHealth) apps providers prescribed to or used with patients stratified by patient age (pediatric vs adult).

Multimedia Appendix 5 Facilitators of adopting mobile health (mHealth) apps in health care settings.

Multimedia Appendix 6 Barriers to adopting mobile health (mHealth) apps in health care settings.

## Abbreviations

CHERRIES: Checklist for Reporting Results of Internet E-Surveys
FDA: Food and Drug Administration
IRB: Institutional Review Board
listserv: list server
mHealth: mobile health
OPENS: Oncology Provider Experiences National Survey
REDCap: Research Electronic Data Capture
RQ: research question
TAM: Technology Acceptance Model
