# Supplementary material for: Mobile Health App Attitudes and Adoption Among Oncology Providers: Cross-Sectional National Survey
Source: J Med Internet Res. 2026 Mar 23;28:e85583. doi: 10.2196/85583 (PMC13054221; doi:10.2196/85583)
Supplement: Multimedia Appendix 2 [file jmir_v28i1e85583_app2.docx]

**Multimedia Appendix 2.** Oncology Provider Experiences National Survey (OPENS): Mobile Health

1. What is your age?
   1. Under 24 years
   2. 24-35 years
   3. 36-45 years
   4. 46-55 years
   5. 56-65 years
   6. 65+ years
2. What is your gender?
   1. Women; Female
   2. Man; Male
   3. Trans feminine
   4. Trans masculine
   5. Nonbinary
   6. Genderqueer
   7. Questioning; Exploring
   8. Gender identity not listed above: ________
   9. Prefer not to respond
3. What is your ethnicity?
   1. Hispanic or Latino
   2. Not Hispanic or Latino
   3. Prefer not to respond
4. What is your race?
   1. American Indian or Alaska Native
   2. Asian
   3. Black or African American
   4. Hispanic or Latino
   5. Middle Eastern or North African
   6. Native Hawaiian or Pacific Islander
   7. White
   8. Race identity not listed above: ________
   9. Prefer not to respond
5. What is your current role within the healthcare system? Select all that apply.
   1. Medical oncologist
   2. Radiation oncologist
   3. Surgical oncologist
   4. Pediatric oncologist
   5. Nurse
   6. Advanced practice provider
   7. Psychologist
   8. Social worker
   9. Healthcare administrator
   10. Researcher
   11. Physician (other specialty): ________
   12. Other: ________
6. What is your practice setting? Select all that apply.
   1. Private practice
   2. Academic/teaching hospital
   3. Community hospital
   4. Hospice care
   5. Other: ________
7. How would you describe your practice location?
   1. Rural
   2. Suburban
   3. Urban
   4. Other: ________
8. How many years have you been providing clinical care for oncology patients?
   1. Less than 1 year
   2. 1-5 years
   3. 6-10 years
   4. 11-15 years
   5. More than 16 years
   6. Prefer not to respond
9. What is the age demographic of oncology patients you typically provide care for? Select all that apply.
   1. Children
   2. Teens
   3. Young Adults
   4. Adults
   5. Older Adults

Q10-Q15. Please indicate your level of agreement with the following statements about mobile health applications. 1 = “Strongly Disagree” to 5 = “Strongly Agree.”

1. I feel confident in my ability to navigate mobile apps.
2. I feel that I am trained and informed about regulatory and ethical issues related to technology use in healthcare.
3. I am comfortable incorporating technology into patient care practices.
4. I can effectively teach patients how to use mobile health technologies for their care.
5. I am interested in receiving more training or specialized training in use of digital tools in my healthcare practice.
6. I can adapt to health technology changes over time.
7. Which types of mobile health applications have you recommended or used with patients? Select all that apply.
   1. Health monitoring and medical symptoms tracking
   2. Medication management
   3. Pain management
   4. Telehealth and virtual consultation
   5. Communication with medical team (including EMR-based messaging apps)
   6. Cancer and disease-management
   7. Exercise and physical activity
   8. Nutritional guidance and diet planning
   9. Mental health and wellness
   10. Sleep health
   11. Health literacy
   12. Social networking and support groups (patient-to-patient interaction)
   13. Energy therapy (e.g. acupressure, reiki, qi gong)
   14. Spiritual care and support
   15. Other: ________
   16. None of the above
8. Which types of mobile health applications are potentially helpful for your patients? Select all that apply.
   1. Health monitoring and medical symptoms tracking
   2. Medication management
   3. Pain management
   4. Telehealth and virtual consultation
   5. Communication with medical team (including EMR-based messaging apps)
   6. Cancer and disease-management
   7. Exercise and physical activity
   8. Nutritional guidance and diet planning
   9. Mental health and wellness
   10. Sleep health
   11. Health literacy
   12. Social networking and support groups (patient-to-patient interaction)
   13. Energy therapy (e.g. acupressure, reiki, qi gong)
   14. Spiritual care and support
   15. Other: ________
   16. None of the above

Q18-Q28. Please rate each statement on mobile technology from 1 = “Completely Disagree” to 5 = “Completely Agree.”

1. The use of mobile technology improves the quality of public health services.
2. Using mobile technology reduces the cost of providing public health services.
3. Mobile technology improves self-management in public healthcare professionals.
4. The use of mobile technology expands the range of health services.
5. The use of mobile technology creates equal access to facilities and services for the general public.
6. Mobile technology improves interpersonal interactions between the provider and the recipient.
7. Using mobile technology reduces the number of visits to health centers.
8. Using mobile technology, travel costs for services are reduced.
9. Mobile technology can help improve patients' quality of life.
10. Mobile technology can improve patients’ health literacy and encourage them to actively engage in their care.
11. Integrating mobile technology can improve patient-provider interactions.
12. Which of the following do you perceive to be facilitators to the adoption of mobile health applications in your healthcare setting? Select all that apply.
    1. Improved job performance and efficiency
    2. Staff monetary incentives
    3. Cost reduction for patients and healthcare systems
    4. Alignment with patients’ expressed interest and needs
    5. Improved patient health literacy
    6. Empowered patient agency and self-management
    7. Improved healthcare accessibility
    8. Demonstrated improvement in patient outcomes
    9. Robust data security and privacy measures
    10. Health institution endorsement
    11. Recommended by government or other regulatory agencies
    12. Recommended by colleagues
    13. Strength of research evidence
    14. Clinician training, ongoing support, and usage guidelines for apps
    15. Seamless integration with existing healthcare systems and/or electronic medical records
    16. Other: ________
    17. None of the above
13. Which of the following do you perceive to be barriers to the adoption of mobile health applications in your healthcare setting? Select all that apply.
    1. Associated costs
    2. Lack of staff monetary incentives
    3. Lack of interest from providers
    4. Lack of interest from patients
    5. Data privacy and safety concerns
    6. Ethical and legal concerns
    7. Disparities in access to technology
    8. Technical usability and lack of technical knowledge
    9. Lack of appropriateness or fit for your patients
    10. Lack of digital use guidelines and technical support
    11. Lack of integration into existing clinical workflows
    12. Time burden for providers
    13. Time burden for patients
    14. Lack of knowledge regarding which digital tools to recommend or where to find relevant information
    15. Overwhelmed by too many digital health technology options
    16. Other: ________
    17. None of the above
