# Supplementary figures and images for: Mobile Health App Attitudes and Adoption Among Oncology Providers: Cross-Sectional National Survey
Source: J Med Internet Res. 2026 Mar 23;28:e85583. doi: 10.2196/85583 (PMC13054221; doi:10.2196/85583)

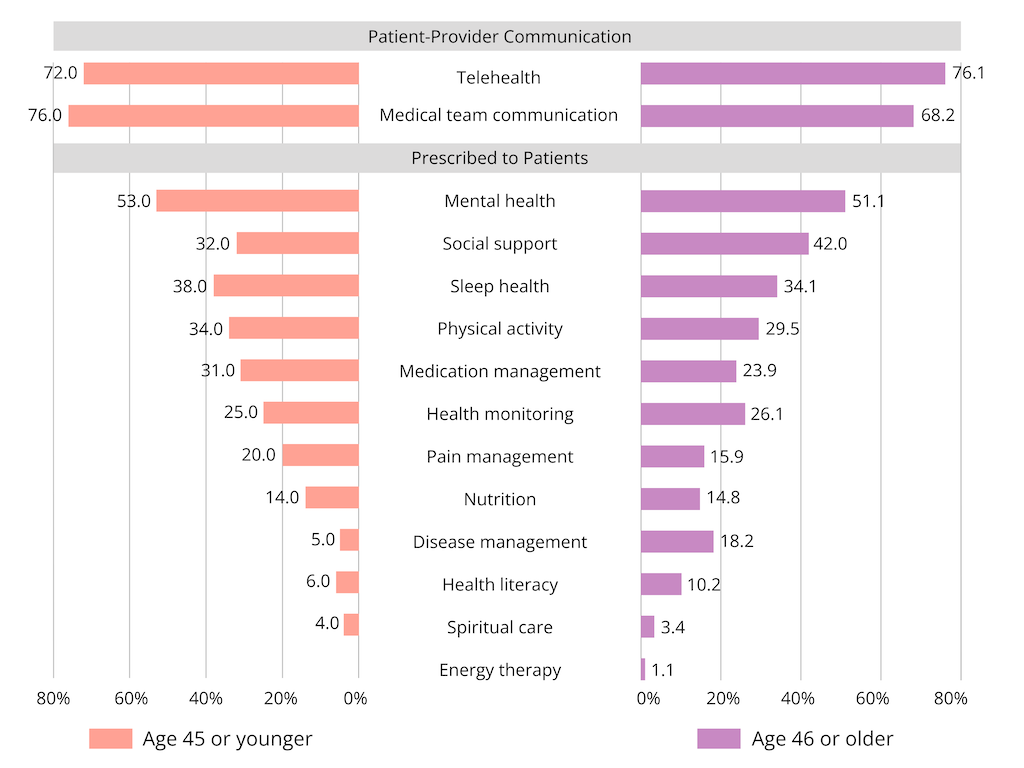

Supplement: Multimedia Appendix 3 [file jmir_v28i1e85583_app3.png]

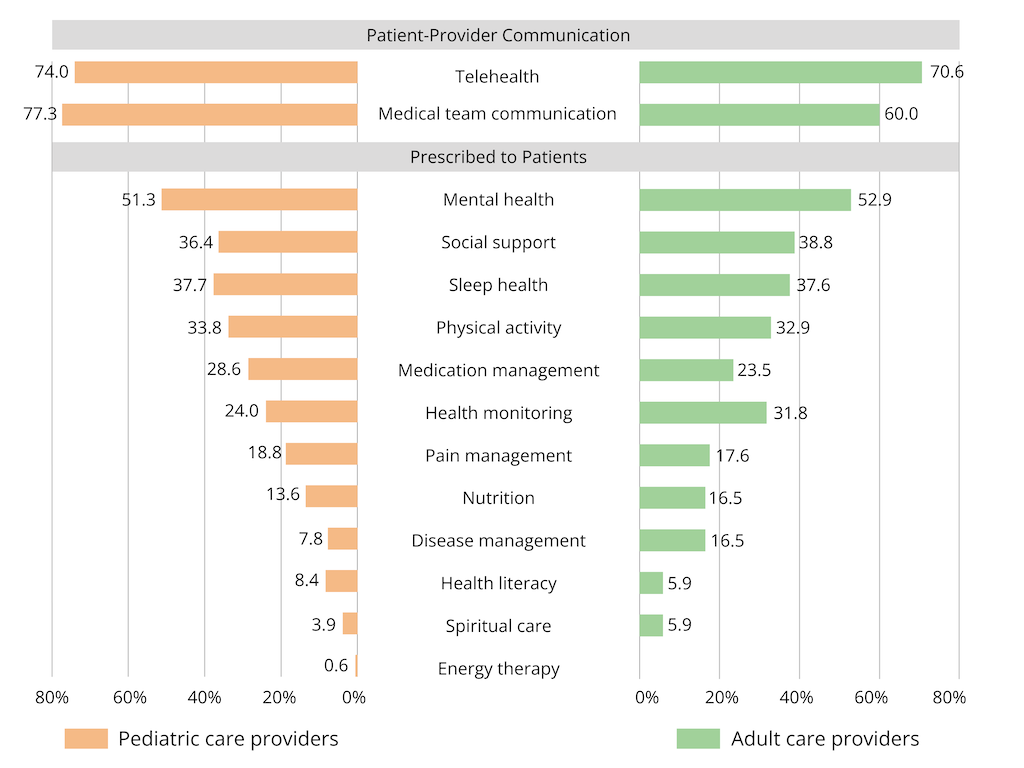

Supplement: Multimedia Appendix 4 [file jmir_v28i1e85583_app4.png]

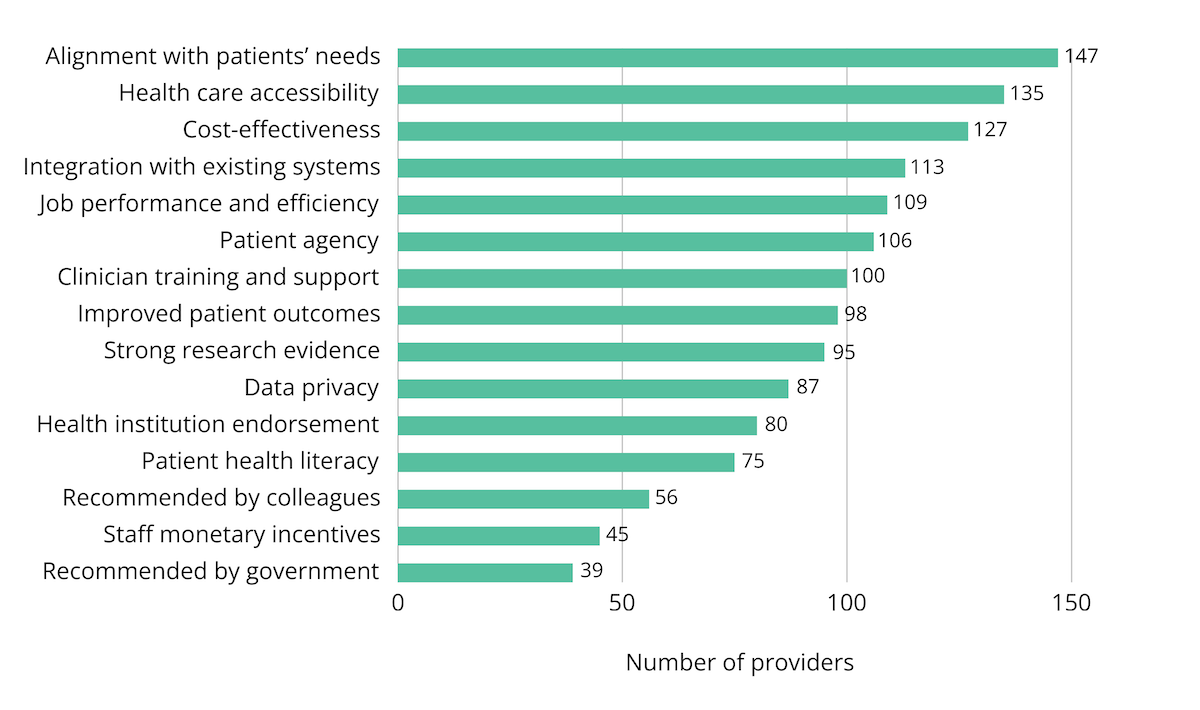

Supplement: Multimedia Appendix 5 [file jmir_v28i1e85583_app5.png]

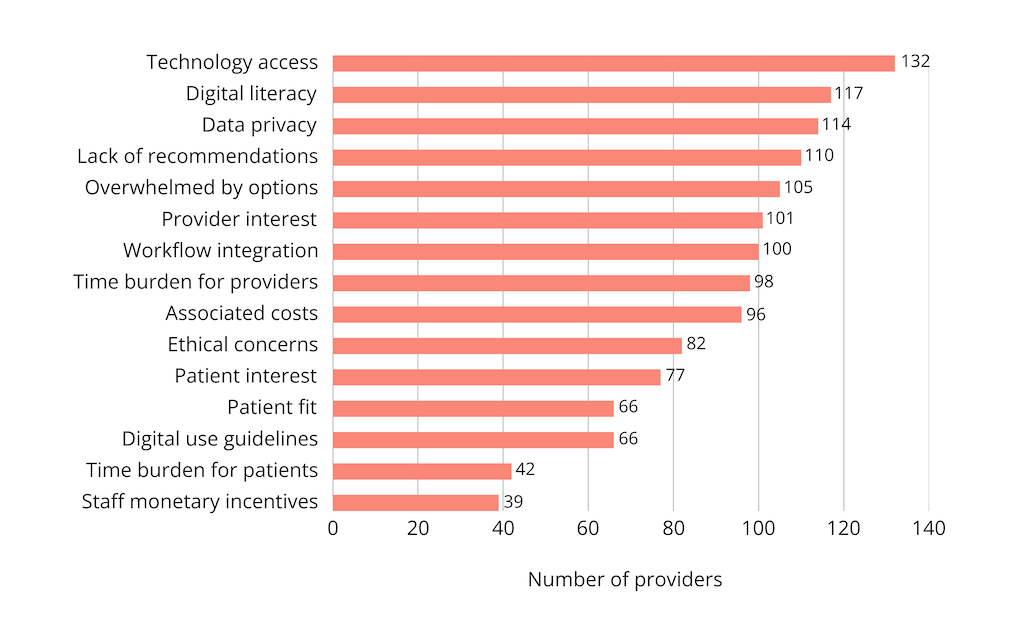

Supplement: Multimedia Appendix 6 [file jmir_v28i1e85583_app6.png]
